# Effect of Nitric Oxide on Adventitious Root Development from Cuttings of Sweetpotato and Associated Biochemical Changes

**DOI:** 10.3390/plants14203183

**Published:** 2025-10-16

**Authors:** Meng Wang, Jianghui Li, Yuhao Wu, Hongxing Zhang, Hui Wang, Lingyun Wang

**Affiliations:** Cotton Research Institute, Shanxi Agricultural University, Yuncheng 044000, China; mkswangmeng@126.com (M.W.); sxcrilijh@126.com (J.L.);

**Keywords:** nitric oxide, adventitious root, sweetpotato, soluble sugar, soluble protein and starch content, rooting-related enzyme activities, chlorophyl content, fluorescence parameters, PSII reaction center parameters

## Abstract

Adventitious rooting is a key step for the clonal propagation of many economically important horticultural and woody species. Accumulating evidence suggests that nitric oxide (NO) serves as a key signaling molecule with key roles in root organogenesis. However, the role of NO in adventitious root development and its underlying mechanism in sweetpotato cuttings remain to be clarified. In this study, a pot experiment was conducted using hydroponically cultured sweetpotato cuttings (*Ipomoea batatas* cv. ‘Jin Ganshu No. 9’) treated with different concentrations of sodium nitroprusside (SNP, an NO donor) solution (0, 10, 50, 100, 200, and 500 μmol·L^−1^). Three treatments were established: Control, SNP (the optimal concentration of SNP), and SNP + 2-(4-carboxyphenyl)-4,4,5,5-tetramethylimidazoline-1-oxyl-3-oxide (cPTIO, an NO scavenger). The results showed that NO promoted adventitious rooting in a dose-dependent manner, with the maximal biological response observed at 100 μM SNP. At this concentration, the root number and length of adventitious roots increased by 1.22 and 2.36 times, respectively, compared to the control. SNP treatment increased fresh root weight, dry root weight, the content of soluble sugar, soluble protein, chlorophyll a (Chl a), chlorophyll b (Chl b), and total chlorophyll (a + b) [Chl(a + b)], as well as the activities of peroxidase (POD), polyphenol oxidase (PPO), and indole acetic acid oxidase (IAAO). It also enhanced the levels of maximum fluorescence (Fm), maximum photochemical efficiency of photosystem II (Fv/Fm), absorbed light energy (ABS/RC), trapped energy flux (TRo/RC), and electron transport flux (ETo/RC), while decreasing starch content and initial fluorescence (Fo). On the 7th day, the SNP treatment significantly enhanced several biochemical parameters compared to the control. We observed an increase in many of the parameters: POD activity by 1.35 times, PPO activity by 0.55 times, chlorophyll content (Chl a by 0.66 times, Chl b by 0.22 times, and Chl a + b by 0.57 times), and photosynthesis parameters by 28–98%. Meanwhile, starch content and Fo in the SNP treatment decreased by 10.77% and 23.86%, respectively, compared to the control. Furthermore, the positive effects of NO on adventitious root development and associated biochemical parameters were reversed by the NO scavenger cPTIO. Additionally, significant and positive correlations were observed between morphological characteristics and most physiological indicators. Collectively, these results demonstrate that NO promotes adventitious root formation, which may be by enhancing rooting-related enzyme activities, improving photosynthetic performance in leaves, and accelerating the metabolism of soluble sugar, soluble protein, and starch.

## 1. Introduction

Sweetpotato (*Ipomoea batatas* Lam.), originating from tropical South America, is widely cultivated in over 120 countries and regions due to its high yield, stability, adaptability, and low maintenance requirements. It is classified by the Food and Agriculture Organization (FAO) as one of the most important root crops globally, essential for food security and energy sustainability [1,2]. China ranks first globally in sweetpotato production and cultivation area, where it is utilized as a key crop for food, feed, industrial raw materials, and renewable energy, following rice, wheat, and maize [3]. Sweetpotato is rich in carbohydrates, sugars, essential nutrients, and antioxidants [4]. In recent years, it has gained traction as a multifunctional health food, driving increased market demand and providing economic benefits to growers while offering significant nutritional value to consumers.

Adventitious roots are post-embryonic roots that develop from non-root organs such as stems, leaves, or hypocotyls, or emerge from wound-healing tissue and stem bases. As essential organs for water and nutrient absorption, as well as assimilate storage, the adventitious root induction and formation are critical for the survival rate of asexually propagated cuttings in horticultural crops [5]. Their development is regulated via complex mechanisms involving endogenous and environmental factors, including phytohormones (auxin, ethylene, and cytokinins), bioactive molecules (hydrogen, nitric oxide, carbon monoxide, and hydrogen sulfide), mechanical wounding, and pathogen infection [6]. Sweetpotato, a typical tuberous root crop propagated vegetatively, primarily reproduces through stem cuttings. Its yield is strongly correlated with root system development [7]. Thus, enhancing adventitious root formation during the cutting process can significantly improve the survival rate of sweetpotato cuttings.

Nitric oxide (NO), a water- and lipid-soluble gaseous signaling molecule, plays extensive roles in plant physiological processes, including seed dormancy [8], hypocotyl elongation [9], flowering [10], and leaf senescence [11]. It also enhances plant tolerance to biotic and abiotic stresses, such as drought [12], salinity [13], and heavy metal toxicity [14]. As a crucial signaling molecule, NO regulates root growth and development through interactions with other endogenous signaling molecules and phytohormones, promoting adventitious root formation in plants such as maize [15], cucumber [16], *Arabidopsis thaliana* [17], and marigold [18]. Li et al. [16] reported that both the number and length of adventitious roots on cucumber explants treated with 200 µM SNP were significantly higher than those treated with distilled water. Despite extensive research on NO in plants over recent decades, the effects of exogenous NO on adventitious root formation in sweetpotato remain poorly understood.

This study utilized the sweetpotato variety ‘Jin Ganshu No. 9’ as the experimental material. Cuttings were treated with sodium nitroprusside (SNP, an NO donor) and 2-(4-carboxyphenyl)-4,4,5,5-tetramethylimidazoline-1-oxyl-3-oxide (cPTIO, an NO scavenger) in a hydroponic cultivation system. Root characteristics were detected, and the dynamics of soluble sugar, soluble protein, and starch content, rooting-related enzyme activities, chlorophyll content, fluorescence parameters, and PSII reaction center parameters were analyzed during adventitious rooting. The research aims to preliminarily elucidate the physiological mechanisms underlying NO-mediated adventitious root formation in sweetpotato, contributing to a deeper understanding of NO signaling in plant growth regulation. The findings may provide a theoretical foundation for advancing high-yield and high-efficiency sweetpotato cultivation.

## 2. Results

### 2.1. Effects of Exogenous SNP on Adventitious Root Development

**Figure 1:**
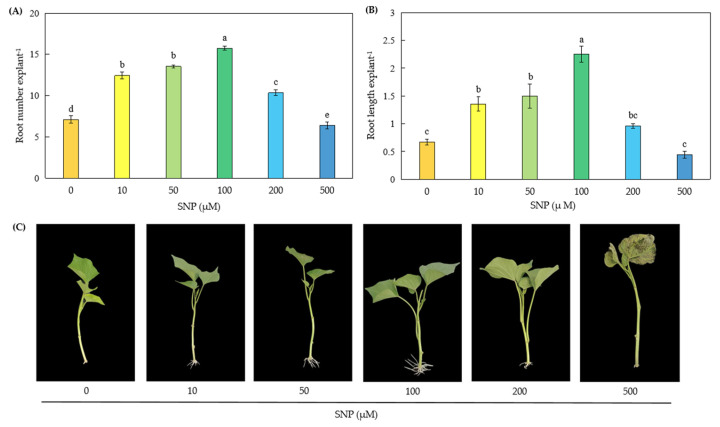
Effects of NO donors SNP on adventitious root development from sweetpotato cuttings. The primary root system was removed from the hypocotyls of 45-day-old sweetpotato seedlings. Cuttings were incubated for 7 d with SNP at 0, 10, 50, 100, 200, and 500 μM, as indicated. The number (**A**) and length (**B**) of adventitious roots per cutting for the six treatments were expressed as mean ± SE (n = 3, with 5 explants per replicate). Different lowercase letters on the bar chart indicate that the difference among treatments is significant (*p* < 0.05) based on one-way ANOVA followed by Duncan’s multiple range test. Photographs (**C**) were taken after 7 d of treatment.

Sweetpotato cuttings were treated with the NO donor SNP at concentrations of 10, 50, 100, 200, and 500 μmol·L^−1^. As shown in Figure 1, SNP significantly influenced adventitious root formation in a concentration-dependent manner. At low concentrations of SNP (10, 50, and 100 μM), the number and length of adventitious roots were markedly increased. Treatments with 200 μM SNP significantly promoted the number of adventitious roots but had no obvious effect on root length. Moreover, 500 μM SNP significantly inhibited adventitious root formation. The most pronounced effects on promoting adventitious root formation were observed at 100 μM SNP, where the number and length of adventitious roots reached (15.75 ± 0.26) and (2.25 ± 0.15) cm, respectively, representing increases of 1.22 and 2.36 times compared to the control (Figure 1). Based on these results, a concentration of 100 μM SNP was selected for subsequent experiments.

### 2.2. Effects of NO Scavenger cPTIO on Adventitious Root Development

**Figure 2:**
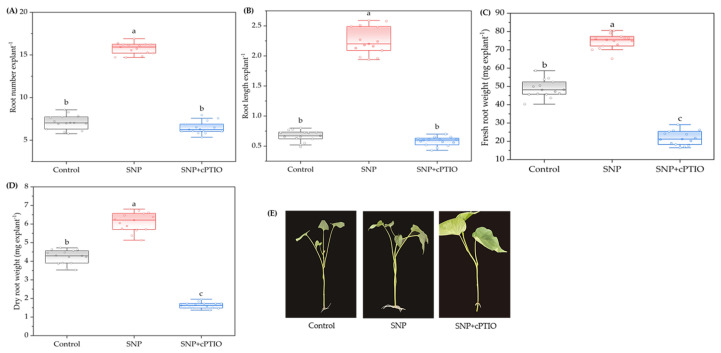
Effects of NO scavenger cPTIO on adventitious root development from sweetpotato cuttings. The primary root system was removed from the hypocotyls of 45-day-old sweetpotato seedlings. Cuttings were incubated for 7 d with distilled water (Control), SNP, and SNP + cPTIO, as indicated. SNP and cPTIO were used at 100 and 200 μM, respectively. Shown are boxplots of root number (**A**), root length (**B**), fresh root weight (**C**), and dry root weight (**D**) for three treatments (n = 3, with 5 explants per replicate). In each plot, the central line represents the median, boxes indicate interquartile ranges, and whiskers show data spread. Different lowercase letters above the boxes indicate that the difference among treatments is significant (*p* < 0.05) based on one-way ANOVA followed by Duncan’s multiple range test. Photographs (**E**) were taken after 7 d of treatment.

To further validate the role of NO in promoting adventitious root formation in sweetpotato, cPTIO (a specific NO scavenger) was employed (Figure 2). The results demonstrated that the promotive effects of SNP (an NO donor) on adventitious root formation were significantly reversed by 200 μM cPTIO, leading to a marked reduction in both the number and length of adventitious roots. Under treatment with SNP + cPTIO, the length and number of adventitious roots decreased by 13.43% and 9.27%, respectively, compared to the control; specifically, these decreases were 74.26% and 59.17%, respectively, compared to the SNP treatment alone (Figure 2A,B,E). Moreover, the fresh and dry weight of adventitious roots were significantly reduced by 55.28% and 61.61%, respectively, under SNP + cPTIO treatment compared with the control treatment (Figure 2C,D). In addition, a supplementary experiment of exogenous cPTIO at different concentrations (0, 100, 200, 300, and 400 µM) on adventitious root development showed that sweetpotato cuttings exhibited normal growth under treatment with 200 µM cPTIO, with no observed symptoms of high-concentration toxicity (Figure S1).

### 2.3. Changes in Soluble Sugar, Soluble Protein, and Starch Contents in Cuttings During Adventitious Rooting

**Figure 3:**
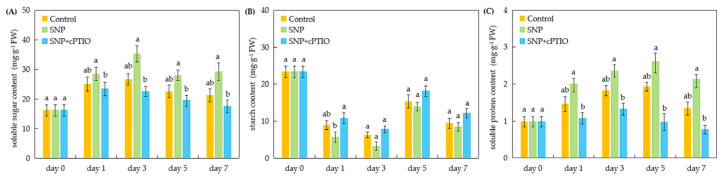
Changes in soluble sugar, soluble protein, and starch content in the rooting region of cuttings during the rooting process. The primary root system was removed from the hypocotyls of 45-day-old sweetpotato seedlings. The contents of soluble sugar (**A**), starch (**B**), and soluble protein (**C**) were determined in cuttings treated with distilled water (Control), SNP, and SNP + cPTIO. SNP and cPTIO were applied at 100 and 200 μM, respectively. Vertical bars represent mean ± SE value (n = 3, with 5 explants per replicate). Different lowercase letters on the bar chart indicate that the difference among treatments on the same day is significant (*p* < 0.05) based on one-way ANOVA followed by Duncan’s multiple range test.

As shown in Figure 3A, the soluble sugar content in sweetpotato cuttings exhibited an initial increase from days 0 to 3, followed by a decline across all treatments from days 3 to 5. Under the SNP treatment, soluble sugar content peaked on day 3, reaching 1.31 times that of the control, and showed a slight increase on day 7. Throughout the experimental period, soluble sugar content in cuttings treated with SNP remained consistently higher than that of the control, although the difference did not reach statistical significance. In contrast, cuttings treated with SNP + cPTIO consistently exhibited lower soluble sugar levels compared to the control, indicating that the promotive effect of SNP on soluble sugar accumulation was counteracted by cPTIO.

As depicted in Figure 3B, the starch content in all treatments exhibited a dynamic pattern: it declined from days 1 to 3, increased from days 3 to 5, and subsequently decreased again from days 5 to 7. Under the SNP treatment, starch content reached its lowest level on day 3, which was 48.57% lower compared to the control. Throughout the experimental period, starch content in SNP-treated cuttings remained consistently lower than that of the control, with no significant difference, a trend that was significantly reversed by cPTIO.

As shown in Figure 3C, the soluble protein content in all treatments displayed an initial increase followed by a decline. Both the SNP treatment and the control peaked on day 5 before decreasing, whereas the SNP + cPTIO treatment peaked earlier on day 3, followed by a rapid decline. At day 5, soluble protein content under the SNP treatment was 35.23% higher compared to the control. Throughout the experiment period, the SNP treatment consistently maintained higher soluble protein levels than the control. However, these differences remained non-significant. Notably, cPTIO significantly attenuated the SNP-induced enhancement of soluble protein content. On day 3, soluble protein content under the SNP + cPTIO treatment was 29.12% lower than the SNP treatment and 27.47% lower than the control.

### 2.4. Changes in Rooting-Related Enzyme Activities in Cuttings During Adventitious Rooting

**Figure 4:**
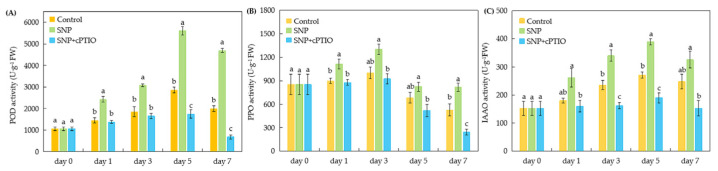
Changes in rooting-related enzyme activities in the rooting region of cuttings during the rooting process. The primary root system was removed from the hypocotyls of 45-day-old sweetpotato seedlings. Peroxidase (POD) activity (**A**), polyphenol oxidase (PPO) activity (**B**), and indole acetic acid oxidase (IAAO) activity (**C**) were determined in cuttings treated with distilled water (Control), SNP, and SNP + cPTIO. SNP and cPTIO were applied at 100 and 200 μM, respectively. Vertical bars represent mean ± SE value (n = 3, with 5 explants per replicate). Different lowercase letters on the bar chart indicate that the difference among treatments on the same day is significant (*p* < 0.05) based on one-way ANOVA followed by Duncan’s multiple range test.

Peroxidase (POD) activity exhibited consistent variations during adventitious root formation in sweetpotato cuttings (Figure 4A). POD activity increased from days 0 to 5, followed by a decline from days 5 to 7 across all treatments. Compared to the control, the SNP treatment showed significantly higher POD activity on days 1, 3, 5, and 7. On day 5, POD activity under the SNP treatment was 1.96 times higher than the control. In contrast, the SNP + cPTIO treatment consistently exhibited lower POD activity than the control, with significant reductions on days 5 and 7. These results indicate that cPTIO effectively suppressed the promotive effect of SNP on POD activity.

As illustrated in Figure 4B, polyphenol oxidase (PPO) activity in all treatments peaked on day 3 and subsequently dropped to lower levels from days 5 to 7. The SNP treatment resulted in significantly higher PPO activity than the control at days 1 and 7, with values 1.24 and 1.55 times greater, respectively. However, the enhancing effect of SNP on PPO activity was markedly inhibited by cPTIO. On day 7, PPO activity under the SNP + cPTIO treatment was significantly lower than the control, showing a 53.50% reduction.

Figure 4C demonstrates that indole acetic acid oxidase (IAAO) activity followed a consistent trend across all treatments, rising steadily from days 0 to 5 before declining from days 5 to 7. The SNP treatment exhibited significantly higher IAAO activity than the control on days 3 and 5, with values 1.45 and 1.44 times greater, respectively. Conversely, cPTIO effectively reduced IAAO activity, resulting in 31.23% and 29.96% lower activity than the control on days 3 and 5, respectively.

### 2.5. Changes in Chlorophyll Content, Fluorescence Parameters, and PSII Reaction Center Parameters in Cuttings During Adventitious Rooting

The content of chlorophyll a (Chl a), chlorophyll b (Chl b), and chlorophyll (a + b) [Chl (a + b)] increased under the SNP treatment and the control but decreased under the SNP + cPTIO treatment during the experiment (Table 1). The contents of Chl a, Chl b, and Chl (a + b) in SNP treatments were significantly higher than those in the control on days 5 and 7. For instance, on day 5, SNP treatment increased the contents of Chl a, Chl b, and Chl (a + b) to levels 31.25%, 21.62%, and 29.28% higher than those in the control, respectively. However, cPTIO significantly reversed the promotive effect of SNP on Chl a content, resulting in a 47.22% decrease on day 5 compared to the control. Similarly, the promoting effects of NO on Chl b and Chl (a + b) contents were counteracted by cPTIO.

Table 2 shows that initial fluorescence (Fo) under the SNP treatment was lower than the control on days 1, 3, 5, and 7. Maximum fluorescence (Fm) of all treatments peaked on day 3 before declining. In contrast to Fo, Fm under the SNP treatment was significantly higher than the control. The maximum photochemical efficiency of photosystem II (Fv/Fm) exhibited obviously different variation tendencies across different treatments. Fv/Fm under the SNP treatment was consistently higher than the control during the experimental period, with a significant increase of 12.16% on day 3. However, the promotive effects of SNP on Fm and Fv/Fm were significantly attenuated by cPTIO. Compared to the SNP treatment and the control, Fv/Fm under the SNP + cPTIO treatment decreased by 79.01% and 74.63% on day 7, respectively.

The absorbed light energy (ABS/RC), trapped energy flux (TRo/RC), and electron transport flux (ETo/RC) reached maximum levels on day 3 before gradually declining (Table 3). Compared to the control, the SNP treatment significantly increased ABS/RC, TRo/RC, and ETo/RC, which were reversed by cPTIO. Specifically, exposure to SNP on day 7 resulted in increases of 47.92% in ABS/RC, 98.10% in TRo/RC, and 28.57% in ETo/RC. In contrast, the SNP + cPTIO treatment resulted in reductions of 35.09%, 22.78%, and 45.71%, respectively.

### 2.6. Correlation Analysis Between Morphological Characteristics and Physiological Indicators

**Figure 5:**
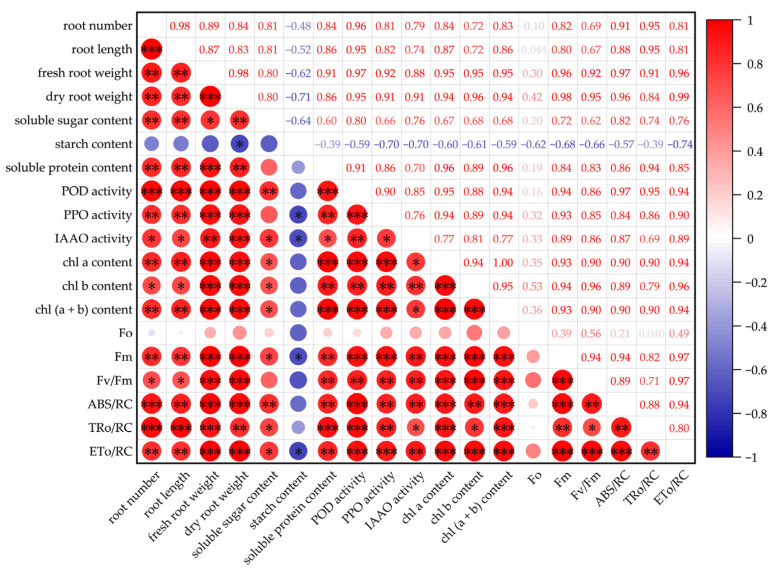
Correlation analysis of root number, root length, fresh root weight, dry root weight, rooting-related enzyme activities, chlorophyll fluorescence, PSII reaction centers parameters, and the contents of soluble sugar, soluble protein, starch, and chlorophyll on day 7 in sweetpotato cuttings. Spearman correlation coefficients were used for the analysis (n = 3, with 5 explants per replicate). Red signifies a positive correlation, while blue indicates a negative correlation. Additionally, an asterisk (*) denotes *p* < 0.05, two asterisks (**) *p* < 0.01, and three asterisks (***) *p* < 0.001.

As shown in Figure 5, the relationship between morphological characteristics and physiological indicators were assessed using Pearson correlation. During adventitious root formation in sweetpotato cuttings, significant and positive correlations were observed between morphological characteristics (root number, root length, fresh root weight, and dry root weight) and most physiological indicators. These physiological indicators included the content of soluble sugar, soluble protein, Chl a, Chl b, and Chl (a + b), the activities of POD, PPO, and IAAO; and the levels of Fm, Fv/Fm, ABS/RC, TRo/RC, and ETo/RC. Specifically, root number and length were highly significantly and positively correlated with POD activity (*p* < 0.001 for both, r = 0.96 and 0.95, respectively) and TRo/RC (*p* < 0.001 for both, r = 0.95 for both). Meanwhile, fresh and dry root weight showed highly significant and positive correlations with POD activity (*p* < 0.001 for both, r = 0.97 and 0.95, respectively), PPO activity (*p* < 0.001 for both, r = 0.92 and 0.91, respectively), Chl a content (*p* < 0.001 for both, r = 0.95 and 0.94, respectively), Chl b content (*p* < 0.001 for both, r = 0.95 and 0.96, respectively), Chl a + b content (*p* < 0.001 for both, r = 0.95 and 0.94, respectively), Fm (*p* < 0.001 for both, r = 0.96 and 0.98, respectively), Fv/Fm (*p* < 0.001 for both, r = 0.92 and 0.95, respectively), ABS/RC (*p* < 0.001 for both, r = 0.97 and 0.96, respectively), and TRo/RC (*p* < 0.001 for both, r = 0.96 and 0.99, respectively). In contrast, dry root weight was significantly negatively correlated with starch content (*p* < 0.05, r = −0.71). Additionally, no significant correlations were observed between morphological characteristics and Fo.

## 3. Discussion

NO is a free radical gas that has biological effects at low concentrations but exerts cytotoxic effects at high concentrations, as previously documented [19]. This study demonstrates that exogenous application of the NO donor SNP significantly enhances the number and length of adventitious roots in sweetpotato cuttings, as illustrated in Figure 1. The effects of SNP were dose-dependent, with the maximal biological response observed at 100 μM. However, at concentrations above 100 μM, the number and length of adventitious roots gradually declined, accompanied by visible injury symptoms such as water loss, wilting, and leaf browning at 500 μM. These adverse effects may result from elevated NO levels inducing oxidative or osmotic stress. Our results are supported by previous studies [20,21,22]. For example, SNP exhibited dual effects on soybean root growth, with low concentrations (5–10 μM) promoting root elongation, while high concentrations (500–1000 μM) inhibited it [23]. Similarly, Liao et al. [24] reported dose-dependent effects of NO on adventitious rooting. Notably, the optimal SNP concentration for root development varies among species. Additionally, the promotive effects of NO on adventitious rooting were significantly suppressed by NO scavengers or inhibitors in species such as *Panax ginseng* [25], cucumber [26], and marigold [27]. Consistent with these findings, our results show that the NO scavenger cPTIO effectively attenuated the promotive effects of SNP on root number, root length, fresh root weight, and dry root weight, confirming the specific role of NO in this process (Figure 2).

A significant amount of soluble sugars and starch is consumed during adventitious root formation, which serves as a primary energy source and reserves to meet the metabolic demands of root development in plant tissues [28,29,30]. Previous studies have demonstrated that NO can significantly alter the content and distribution of soluble sugars and starch in plants [31,32,33]. Similarly, our results indicate that exogenous NO markedly promoted adventitious root formation in sweetpotato cuttings, possibly accompanied by changes in soluble sugar and starch contents (Figure 3A,B). Higher sugar concentrations have been shown to stimulate adventitious root formation in Arabidopsis, while lower concentrations inhibit it in poplar [34,35]. In our study, the soluble sugar content in SNP-treated cuttings was consistently higher than in the control throughout the rooting period (Figure 3A). Liu et al. [36] reported an 11.2% increase in soluble sugar accumulation during adventitious root formation with SNP treatment. Notably, starch content exhibited the opposite trend, with SNP-treated cuttings maintaining lower levels compared to the control (Figure 3B). This aligns with findings in the rice mutant *plr1*, where reduced starch accumulation in the basal shoot and increased sugar content in the root were closely associated with enhanced lateral root development [32]. According to Fishel et al. [37], starch can be converted into soluble sugars at specific growth stages, providing essential energy for adventitious root formation. These findings suggest that NO may regulate adventitious root formation in sweetpotato cuttings by modulating the interconversion between starch and soluble sugars. Proteins are also regarded as key factors facilitating adventitious root formation in plants [38]. SNP, as an NO donor, has been shown to increase protein content and improve plant growth and physiological functions in wheat [39], pepper [40], and *Brassica rapa* [41] under abiotic stress conditions. In our study, the soluble protein content in cuttings treated with SNP remained higher than that of the control throughout the experiment (Figure 3C). The results were consistent with those reported by Liao et al. [21], who demonstrated that exogenous NO increased soluble protein content and promoted adventitious root development in marigold explants. However, the NO scavenger cPTIO suppressed the positive effects of NO on soluble sugars, starch, and protein content (Figure 3). Moreover, the root number, root length, fresh root weight, and dry root weight were highly and positively correlated with soluble sugar and soluble protein content, respectively. Dry root weight was significantly negatively correlated with starch content (Figure 5). These results further confirm the critical roles of soluble sugars, starch, and proteins in NO-induced adventitious root formation. Thus, our findings suggest that the enhancement of adventitious rooting by NO may be associated with its promotion of starch hydrolysis and the accumulation of soluble sugar and soluble protein.

It is well established that adventitious root formation involves the catalytic activity of multiple enzymes [42]. Growing evidence suggests that POD, PPO, and IAAO play critical roles in the rooting process of cuttings across various plant species [43,44,45]. In this study, the activities of POD, PPO, and IAAO were consistently higher in SNP-treated sweetpotato cuttings compared to the control during both the induction and growth stages (Figure 4). Similarly, root growth of several crop species, including wheat [46], mung bean [47], ginseng [25], and tomato [48], has been closely associated with NO-induced enhancements in the activities of POD and PPO. For instance, Sharma et al. [47] reported that exogenously applied NO significantly enhanced SOD and POD activities in the hypocotyls of *Vigna radiata* during adventitious root formation, with POD activity increasing by 11% and 17% on the 3rd and 5th days of 0.5 µM SNP treatment, respectively. These findings align with those of Liao et al. [49], who demonstrated that SNP treatment in ground-cover chrysanthemum enhanced the activities of PPO, POD, and IAAO during adventitious rooting. Importantly, POD, PPO, and IAAO are closely associated with auxin metabolism, highlighting their pivotal role in regulating adventitious root development. In particular, IAAO is a key enzyme responsible for degrading indole-3-acetic acid (IAA), thereby promoting adventitious roots formation by modulating IAA levels in plants [50,51]. These findings suggest that NO could promote adventitious root formation in cuttings by enhancing the activities of POD, PPO, and IAAO, which may be associated with an increase in the levels of free auxin. Interestingly, our results are not entirely consistent with those of Liu et al. [36], who reported that SNP treatment in tomato enhanced the activities of PPO and POD during adventitious rooting but reduced IAAO activity. This discrepancy highlights that the synergistic network between NO and rooting-related enzymes is complex and diverse during adventitious root development and may vary significantly across different plant species. Furthermore, the NO scavenger cPTIO significantly mitigated the increases in POD, PPO, and SOD activities induced by NO (Figure 4). Similar declines in POD and PPO activities were observed in mung bean and tomato during NO-induced adventitious rooting when treated with cPTIO [36,47], consistent with the results of our study. Previous studies have shown that the interaction between SNP and phytohormones, particularly auxin, is important in regulating root growth [52,53]. Wei et al. [54] demonstrated that SNP treatment affected various auxin-mediated processes, including root elongation, lateral root development, and apical dominance through alterations in auxin concentrations and distribution. Similar results were reported by Chavoushi et al. [55], who found SNP treatment could enhance primary root elongation, an effect attributed to SNP-induced regulation of auxin levels and distribution. Moreover, Jin et al. [27] also reported that NO might be involved in ethylene-induced adventitious root development, which enhanced adventitious root development by stimulating the activities of IAAO, POD, and PPO. Additionally, root number, root length, fresh root weight, and dry root weight showed a significantly positive correlation to the activities of POD, PPO, and IAAO (Figure 5). These findings further demonstrate that NO may catalyze auxin metabolism by enhancing the activities of POD, PPO, and IAAO, thereby promoting adventitious root formation in sweetpotato cuttings.

Chlorophyll, a key component of the plant photosynthetic apparatus, plays a pivotal role in light energy absorption, transmission, and conversion [56]. Numerous studies have demonstrated that NO participates in the plant growth and development process by mediating and regulating chlorophyll metabolism [57,58,59]. According to our results (Table 1), the content of total chlorophyll (Chl a + b), Chl a, and Chl b under SNP treatment were higher than those in the control, particularly during the root elongation stage. Similar findings have been reported in previous studies, where SNPs significantly boosted chlorophyll levels in seedlings of peanut [59], wheat [60], and citrus [61]. These results align with the findings that SNPs retarded the degradation of chlorophyll in marigold and cucumber explants during adventitious root development [21,62]. Moreover, NO has been previously associated with iron (Fe) homeostasis in plants [63,64]. The application of SNP enhanced the translocation of Fe from roots to shoots and its transfer from the cell wall to the organelle and soluble fractions, thereby increasing the available Fe in cell organelles, and the active Fe, chlorophyll contents in peanut leaves [65]. This is consistent with the results of Graziano and Lamattina [66], who reported that the enhanced ability of SNP-treated plants to augment chlorophyll content may be attributed to an increase in the availability of endogenous iron. It has been shown that Fe serves not only as a constituent of chloroplast pigments but also plays an essential role in the electron transport chain during plant photosynthesis [67]. Taken together, these previous studies imply the possibility that the SNP treatment enhances chlorophyll content during adventitious root formation in sweetpotato cuttings, potentially through a mechanism involving increased iron availability. Nonetheless, the precise mechanism through which NO promotes chlorophyll biosynthesis remains to be fully elucidated. Thus, these studies highlight the role of NO in enhancing chlorophyll content and stability, which may contribute to improved photosynthetic efficiency and overall plant growth during adventitious root formation. Chlorophyll fluorescence parameters serve as ideal probes for studying plant photosynthetic physiology, reflecting the photosynthetic capacity and environmental adaptability of plants to a certain extent [68]. A decrease in NO content resulted in a reduction of Fv/Fm in *Arabidopsis* seedlings overexpressing spinach non-symbiotic hemoglobin, indicating that NO levels are closely related to the functional integrity of photosystem II (PSII) [69]. While numerous studies have reported the effects of NO on plant fluorescence parameters under various stress conditions [70,71,72], relatively few investigations have examined these effects under non-stress conditions. For example, SNP significantly inhibited the apparent decline in Fv/Fm and the obvious increase in Fo under enhanced UV-B radiation in *Larix gmelinii* [73]. Similarly, treatment with 0.1 mmol/L SNP maintained higher Fv/Fm and lower Fo in wheat leaves under high temperature and strong irradiance stress [74]. Additionally, NO-treated peanut exhibited higher Fv/Fm and lower Fo under iron-deficiency chlorosis [59]. In this study, we verified that SNP increased Fo, Fm, and Fv/Fm in sweetpotato leaves under non-stress conditions (Table 2). Ma et al. [75] proposed that an elevated ABS/RC may be beneficial for maintaining Fv/Fm stability under severe drought stress. In our study, parameters of energy fluxes, including ABS/RC, TRo/RC, and ETo/RC, were enhanced in SNP-induced cuttings compared to the control (Table 3). These results contrast with those of Verma and Prasad [76], who reported a sharp drop in ABS/RC, TRo/RC, and ETo/RC in mustard seedlings upon SNP addition under arsenic-induced toxicity. The observed differences could be attributed to variations in the environmental conditions under which the plants were cultivated. More importantly, root number, root length, fresh root weight, and dry root weight exhibited a strong positive correlation with the values of Fm, Fv/Fm, ABS/RC, TRo/RC, and ETo/RC (Figure 5). Notably, the positive impacts of SNP on these parameters were diminished by PTIO, highlighting the importance of NO in sustaining the overall PSII photochemistry.

## 4. Materials and Methods

### 4.1. Plant Material and Growth Conditions

Healthy and uniform stem cuttings of sweetpotato (*Ipomoea batatas* cv. ‘Jin Ganshu No. 9’), measuring 12–15 cm in length and 4.5 mm in diameter, were collected from the basal portion of 45-day-old seedlings at the experimental base of the Cotton Research Institute, Shanxi Agricultural University (Yuncheng, China). Immediately after collection, the cuttings were placed in sealed plastic bags lined with moistened paper towels and stored at 4 °C. For hydroponic culture, the cuttings were transferred to pots containing distilled water or treatment solutions, with five cuttings per pot, and maintained in a growth chamber for 7 days. The growth chamber was set to 25 ± 1 °C with a 14-h photoperiod (photosynthetically active radiation = 200 μmol s^−1^ m^−2^). Treatment solutions were replenished every 24 h to ensure consistent conditions.

### 4.2. Chemicals and Treatments

Sodium nitroprusside (SNP; Beijing Solarbio Science and Technology Co., Ltd., Beijing, China) was used as an NO donor, and 2-(4-carboxyphenyl)-4,4,5,5-tetramethylimidazoline-1-oxyl-3-oxide (cPTIO; Shanghai Aladdin Biochemical Technology Co., Ltd., Shanghai, China) was employed as an NO scavenger. A 500 mM SNP stock solution was prepared in complete darkness and immediately diluted to the desired concentrations. The solutions were stored in amber bottles at 4 °C in the dark, and the interval between preparation and use did not exceed 48 h. The test solutions included (1) different concentrations of SNP (0, 10, 50, 100, 200, and 500 μM); (2) different concentrations of cPTIO (0, 100, 200, 300, and 400 μM); (3) 100 μM SNP +200 μM cPTIO. Each experiment was repeated 3 times with 5 replicates. A total of approximately 810 cuttings were utilized in all experiments. These cuttings were uniformly allocated as follows: 255 for morphological characteristics measurement, 540 for determination of physiological indicators, and 15 for photographic documentation.

### 4.3. Morphological Detection of Adventitious Roots

Any cutting that had at least one root was classified as rooted. The data collected included root number per cutting, root length per cutting, fresh root weight per cutting, and dry root weight per cutting. Adventitious root length was measured using an electronic digital caliper (0–150 mm), and roots longer than 1 mm for each explant were counted. Explants were removed from the petri dish, rinsed the surface of the seedlings with deionized water, and wiped clean with filter paper to remove any remaining solution. For dry weight determination, the dried explants were placed in an electric blast constant temperature oven (Yiheng, Shanghai, China) at 60–80 °C for 24–48 h until a constant weight was achieved [77]. Fresh root weight and dry root weight per cutting were measured using a BS124S electronic balance (Sartorius, Göttingen, Germany). Observations on these morphological characteristics were recorded 7 d after hydroponic cultivation.

### 4.4. Measurement of Soluble Sugar, Soluble Protein, and Starch

Sweetpotato cuttings were subjected to different treatments for 0, 1, 3, 5, and 7 d. After treatment, stem segments within 2 cm of the base of the cuttings were collected and analyzed for soluble sugar, soluble protein, and starch.

Soluble sugar content was determined using the anthrone method, following the procedures of van Handel [78] with slight modifications. Distilled water was used as the control. Reagent blanks were prepared by mixing 2 mL of water, 0.5 mL of anthrone solution, and 5 mL of concentrated sulfuric acid. Sweetpotato cuttings (0.2 g) were cut into pieces and placed into stoppered test tubes, and 5 mL of distilled water was added to each tube. The extraction was performed twice, each for 30 min in boiling water. The extract solution was filtered into a 25 mL volumetric flask, and the test tube and residue were rinsed repeatedly to reach the volume mark. A 0.5 mL aliquot of the extract solution was mixed with 1.5 mL of distilled water, 0.5 mL of anthrone reagent, and 5.0 mL of sulfuric acid (98%) in a test tube. The mixture was heated in a boiling water bath for 1 min, then cooled to room temperature. Using the blank as a reference, the absorbance was measured at 630 nm, and the soluble sugar concentration was determined using a standard curve.

Soluble protein content was quantified using the Coomassie Brilliant Blue method, adapted from Bradford [79] with minor modifications. Sweetpotato cuttings (0.2 g) were ground and extracted in 5 mL of distilled water. The supernatant was obtained through centrifugation at 4 °C and 4000 g for 10 min. The supernatant was then diluted to a final volume of 10 mL. A 1.0 mL aliquot of the sample extract was mixed with 5 mL of Coomassie Brilliant Blue G-250 solution and thoroughly mixed. After standing for 2 min, the absorbance was measured at 595 nm, and protein concentration was determined using a standard curve (the control: 1 mL of distilled water mixed with 5 mL of Coomassie Brilliant Blue G-250 solution). Protein concentrations were determined using a standard curve generated with bovine serum albumin (BSA).

Starch content was measured by homogenizing 0.2 g of sweetpotato cuttings with 2 mL of distilled water and 3.2 mL of 60% HClO_4_. The mixture was adjusted to a final volume of 100 mL with distilled water and centrifuged at 5000 rpm for 5 min. A 0.5 mL aliquot of the supernatant was diluted with distilled water to a total volume of 3 mL. Next, 2 mL of iodine reagent was added, mixed thoroughly, and left to stand for 5 min. Finally, 10 mL of distilled water was added, and the optical density (OD) was measured at 660 nm using a control as a reference. A standard curve generated with known concentrations of soluble starch was used to determine starch concentration in the samples [80].

### 4.5. Measurement of Rooting-Related Enzyme Activities

The sampling time and detection site for determining rooting-related enzyme activities were identical to those used for soluble sugar content measurement.

Fresh tissue (0.2 g) was homogenized in 1.6 mL of 50 μM phosphoric buffer (pH 7.8) using a chilled pestle and mortar, followed by centrifugation at 4000 g for 30 min. The supernatant collected was used as the enzyme extract.

For peroxidase (POD) activity, 0.1 mL of enzyme extract was added to 3 mL of substrate mixture containing 1 mL of 0.1 μM potassium phosphate buffer (pH 7.8), 2% guaiacol, 0.95 mL of 2% guaiacol, and 3 mL of 3% H_2_O_2_. A buffer solution was used as a control instead of the enzyme solution, and the rate of increase in optical density (OD) at 470 nm was measured [81].

For polyphenol oxidase (PPO) activity, 0.1 mL of enzyme extract was added to 5 mL of substrate mixture containing 3.9 mL of phosphate buffer (pH 6.0) and 1 mL of 1 mM pyrocatechol. After mixing, the mixture was incubated at 30 °C for 10 min, and 2 mL of 20% trichloroacetic acid was added to terminate the reaction. The absorbance was immediately measured at 525 nm [81].

To determine indole acetic acid oxidase (IAAO) activity, a reaction mixture [1 mL of enzyme extract solution, 5 mL of potassium phosphate buffer (pH 6.0), 2 mL of 1 mM MnCl_2_, 1 mL of 1 mM 2,4-dichlorophenol, and 2 mL of 1 mM IAA] was incubated at 30 °C for 30 min. Subsequently, 2 mL of the reaction solution was transferred from each test tube to another test tube. A 4 mL reaction solution (containing 1.0 mL of 0.5 M FeCl_3_ and 3 mL of 35% perchloric acid) was added to each test tube and mixed in a dark environment. The degradation of IAA was determined by measuring the absorbance at 535 nm after 30 min [82].

### 4.6. Measurement of Chlorophyll Content

Mature leaves from the same position of each sweetpotato seedling were cut into 0.2 cm pieces and thoroughly mixed. A 0.15 g sample of the mixed sweetpotato leaves was placed in a 25 mL tube. After adding 0.5 mL of acetone, the volume was adjusted to 15 mL with 80% acetone to fully immerse the leaves. Tubes were kept at room temperature in the dark for 24 h. When the leaves turned white, the volume was adjusted to 25 mL with 80% acetone, and absorbance was measured at 665 nm, 649 nm, and 470 nm, respectively [83]. Chlorophyll content was expressed in mg g^−1^ fresh weight.

### 4.7. Measurement of Chlorophyll Fluorescence

Chlorophyll fluorescence parameters of sweetpotato leaves were measured on days 0, 1, 3, 5, and 7 after hydroponic cultivation using a Palmtop chlorophyll fluorescence spectrometer (FluorPen 110, Beijing Ecological Technology Ltd., Beijing, China) at 25 °C. The intensities of the modulated measuring beam, actinic light, and saturating light were set to 0.1 µmol m^−2^ s^−1^, 81 µmol m^−2^ s^−1^, and 2700 µmol m^−2^ s^−1^ PFD, respectively, as per the manufacturer’s instructions. Saturation pulses were applied for 0.8 s, and actinic light was maintained for 5 min to achieve steady-state chlorophyll fluorescence. Following a 30-min dark adaptation period, the initial fluorescence yield (Fo), maximum fluorescence yield (Fm), absorbed light energy (ABS/RC), trapped energy flux (TRo/RC), and electron transport flux (ETo/RC) were recorded. Variable chlorophyll fluorescence (Fv) was evaluated as Fv = Fm − Fo, and the maximum quantum yield of photosystem II (Fv/Fm) was derived as Fv/Fm = (Fm − Fo)/Fm.

### 4.8. Statistical Analysis

The data presented in the figures and tables are expressed as mean ± standard error (SE) from three replicates for each sample. Data collation was carried out using Microsoft Excel 2010 software. Statistical methods were performed using one-way analysis of variance (ANOVA) to examine root number, root length, fresh root weight, dry root weight, rooting-related enzyme activities, chlorophyll fluorescence, and the content of soluble sugar, soluble protein, starch, and chlorophyll. The difference (Duncan, at a 0.05 probability level) test was subsequently applied in SPSS 22.0 (SPSS Inc., Chicago, IL, USA) to determine significant differences among the treatments on the same day. Pearson’s correlation analysis was conducted on measurements from the three treatments (Control, SNP, and SNP + cPTIO) and calculated values at a significance level of *p* < 0.05, *p* < 0.01, and *p* < 0.001, all of which satisfied the normality assumption. Graphical presentation was generated using OriginPro 2025 (OriginLab Corporation, Northampton, MA, USA).

## 5. Conclusions

In conclusion, optimal concentrations of SNP promoted adventitious root development in sweetpotato cuttings. SNP, at the appropriate dosage, increased the content of soluble sugars, soluble proteins, and chlorophyll, as well as fluorescence parameters and PSII reaction center parameters, while enhancing the activities of POD, PPO, and IAAO and reducing starch content and Fo. These effects of SNP were reversed by cPTIO, highlighting the specific role of NO in these processes. Root number, root length, fresh root weight, and dry root weight were significantly positively correlated with the contents of soluble sugars, soluble proteins, Chl a, Chl b, and Chl (a + b); the activities of POD, PPO, and IAAO; and the values of Fm, Fv/Fm, ABS/RC, TRo/RC, and ETo/RC. Dry root weight was significantly negatively correlated with starch content. Collectively, these findings demonstrate that NO-induced adventitious rooting may be achieved by stimulating the content of soluble sugar, soluble protein, starch, and chlorophyll; increasing the activities of key enzymes; improving energy flux parameters; and reducing starch levels. This study provides valuable insights into the role of NO in adventitious root development in cuttings. Future research should focus on elucidating the molecular mechanisms underlying NO regulation of adventitious root formation, which will further advance our understanding of this complex physiological process.

## Figures and Tables

**Table 1:** Changes in chlorophyll content in cuttings during the adventitious rooting process.

Time (Day)	Treatments	Chl a Content (mg·g^−1^ FW)	Chl b Content (mg·g^−1^ FW)	Chl (a + b) Content (mg·g^−1^ FW)
0	Control	0.92 ± 0.11 a	0.32 ± 0.02 a	1.24 ± 0.13 a
	SNP	0.93 ± 0.17 a	0.30 ± 0.02 a	1.23 ± 0.19 a
	SNP + cPTIO	0.96 ± 0.11 a	0.31 ± 0.01 a	1.27 ± 0.16 a
	Control	1.04 ± 0.12 a	0.33 ± 0.02 a	1.37 ± 0.10 ab
1	SNP	1.30 ± 0.16 a	0.34 ± 0.02 a	1.64 ± 0.14 a
	SNP + cPTIO	0.90 ± 0.04 a	0.29 ± 0.02 a	1.19 ± 0.03 b
	Control	1.22 ± 0.13 ab	0.36 ± 0.02 a	1.58 ± 0.14 a
3	SNP	1.60 ± 0.10 a	0.40 ± 0.01 a	2.00 ± 0.09 a
	SNP + cPTIO	0.84 ± 0.05 b	0.26 ± 0.02 b	1.10 ± 0.06 b
	Control	1.44 ± 0.09 b	0.37 ± 0.02 b	1.81 ± 0.07 b
5	SNP	1.89 ± 0.09 a	0.45 ± 0.02 a	2.34 ± 0.10 a
	SNP + cPTIO	0.76 ± 0.06 c	0.22 ± 0.01 c	0.98 ± 0.05 c
	Control	1.55 ± 0.25 b	0.41 ± 0.02 b	1.96 ± 0.27 b
7	SNP	2.57 ± 0.17 a	0.50 ± 0.02 a	3.07 ± 0.18 a
	SNP + cPTIO	0.53 ± 0.02 c	0.12 ± 0.02 c	0.65 ± 0.04 c

Note: The primary root system was removed from the hypocotyls of 45-day-old sweetpotato seedlings. The contents of chlorophyll a (Chl a), chlorophyll b (Chl b), and total chlorophyll (a + b) [Chl (a + b)] were determined in cuttings treated with distilled water (Control), SNP, and SNP + cPTIO. SNP and cPTIO were applied at 100 and 200 μM, respectively. Values (mean ± SE) are the average of three independent experiments with five replicates each. Values not sharing the same letters in a column within CK, SNP, or SNP + cPTIO treatment were significantly different according to one-way ANOVA followed by Duncan’s multiple range test.

**Table 2:** Changes in fluorescence parameters in cuttings during the adventitious rooting process.

Time (Day)	Treatments	Fo	Fm	Fv/Fm
0	Control	9842.67 ± 373.51 a	30,173.67 ± 954.54 a	0.67 ± 0.01 a
	SNP	10,718.00 ± 872.97 a	33,011.33 ± 1539.85 a	0.68 ± 0.01 a
	SNP + cPTIO	10,678.33 ± 1823.33 a	28,300.67 ± 1456.82 a	0.62 ± 0.06 a
	Control	10,982.67 ± 57.78 a	39,947.33 ± 454.67 b	0.73 ± 0.003 a
1	SNP	10,942.67 ± 290.80 a	58,755.33 ± 2325.39 a	0.81 ± 0.003 ab
	SNP + cPTIO	13,084.67 ± 1062.17 a	32,498.00 ± 3182.41 b	0.58 ± 0.08 b
	Control	11,807.67 ± 1064.87 a	45,558.33 ± 1447.70 b	0.74 ± 0.02 a
3	SNP	11,443.33 ± 538.37 a	67,368.33 ± 1337.61 a	0.83 ± 0.01 b
	SNP + cPTIO	13,193.67 ± 198.59 a	40,107.67 ± 2498.11 c	0.67 ± 0.02 b
	Control	12,340.00 ± 192.16 a	43,876.33 ± 731.07 a	0.72 ± 0.01 a
5	SNP	12,053.33 ± 847.66 a	57,320.67 ± 1509.85 b	0.79 ± 0.03 a
	SNP + cPTIO	14,000.00 ± 977.05 a	31,314.67 ± 2506.67 c	0.55 ± 0.06 b
	Control	13,702.67 ± 1691.16 ab	41,514.00 ± 2295.77 a	0.67 ± 0.04 a
7	SNP	10,433.33 ± 981.46 b	54,955.67 ± 2234.24 b	0.81 ± 0.02 a
	SNP + cPTIO	19,526.70 ± 1612.81 a	23,603.88 ± 1326.81 c	0.17 ± 0.07 b

Note: The primary root system was removed from the hypocotyls of 45-day-old sweetpotato seedlings. Initial fluorescence (Fo), maximum fluorescence (Fm), and maximum photochemical efficiency of photosystem II (Fv/Fm) were determined in cuttings treated with distilled water (Control), SNP, and SNP + cPTIO. SNP and cPTIO were applied at 100 and 200 μM, respectively. Values (mean ± SE) are the average of three independent experiments with five replicates each. Values not sharing the same letters in a column within CK, SNP, or SNP + cPTIO treatments were significantly different according to one-way ANOVA followed by Duncan’s multiple range test.

**Table 3:** Changes in PSII reaction centers parameters in cuttings during the adventitious rooting process.

Time (Day)	Treatments	ABS/RC	TRo/RC	ETo/RC
0	Control	2.90 ± 0.07 a	1.95 ± 0.03 a	0.35 ± 0.01 a
	SNP	2.59 ± 0.28 a	1.88 ± 0.05 a	0.36 ± 0.04 a
	SNP + cPTIO	3.01 ± 0.46 a	1.82 ± 0.11 a	0.35 ± 0.03 a
	Control	3.57 ± 0.05 b	2.36 ± 0.29 b	0.46 ± 0.02 b
1	SNP	4.56 ± 0.21 a	3.73 ± 0.11 a	0.64 ± 0.03 a
	SNP + cPTIO	3.07 ± 0.10 b	2.03 ± 0.19 b	0.36 ± 0.02 c
	Control	3.58 ± 0.16 b	2.42 ± 0.34 b	0.55 ± 0.02 b
3	SNP	4.63 ± 0.26 a	4.27 ± 0.38 a	0.78 ± 0.02 a
	SNP + cPTIO	3.16 ± 0.17 b	2.29 ± 0.32 b	0.45 ± 0.01 c
	Control	3.01 ± 0.14 b	2.08 ± 0.16 b	0.42 ± 0.001 ab
5	SNP	4.29 ± 0.17 a	3.66 ± 0.12 a	0.54 ± 0.08 a
	SNP + cPTIO	2.52 ± 0.07 b	1.78 ± 0.05 b	0.27 ± 0.02 b
	Control	2.65 ± 0.17 b	1.58 ± 0.13 b	0.35 ± 0.02 b
7	SNP	3.92 ± 0.20 a	3.13 ± 0.23 a	0.45 ± 0.02 a
	SNP + cPTIO	1.72 ± 0.07 c	1.22 ± 0.10 b	0.19 ± 0.01 c

Note: The primary root system was removed from the hypocotyls of 45-day-old sweetpotato seedlings. Absorbed light energy (ABS/RC), trapped energy flux (TRo/RC), and electron transport flux (ETo/RC) were determined in cuttings treated with distilled water (Control), SNP, and SNP + cPTIO. SNP and cPTIO were applied at 100 and 200 μM, respectively. Values (mean ± SE) are the average of three independent experiments with five replicates each. Values not sharing the same letters in a column within CK, SNP or SNP + cPTIO treatment were significantly different according to one-way ANOVA followed by Duncan’s multiple range test.

## Data Availability

All data generated or analyzed during this study are included in this published article.

## Supplementary Materials

The following supporting information can be downloaded at https://www.mdpi.com/article/10.3390/plants14203183/s1. Figure S1: Effects of the NO scavenger cPTIO on adventitious root development in sweetpotato cuttings.

## Abbreviations

The following abbreviations are used in this manuscript:

NO	Nitric oxide
SNP	Sodium nitroprusside
cPTIO	2-(4-carboxyphenyl)-4,4,5,5-tetramethylimidazoline-1-oxyl-3-oxide
POD	Peroxidase
PPO	Polyphenol oxidase
IAAO	Indole acetic acid oxidase
Chl a	Chlorophyll a
Chl b	Chlorophyll b
Chl (a + b)	Chlorophyll (a + b)
Fo Fm	Initial fluorescence Maximum fluorescence
Fv/Fm	Maximum photochemical efficiency of photosystem II
ABS/RC	Absorbed light energy
TRo/RC	Trapped energy flux
ETo/RC	Electron transport flux
