# Supplementary material for: Effect of Nitric Oxide on Adventitious Root Development from Cuttings of Sweetpotato and Associated Biochemical Changes
Source: Plants (Basel). 2025 Oct 16;14(20):3183. doi: 10.3390/plants14203183 (PMC12567240; doi:10.3390/plants14203183)
Supplement: Supplementary file 1 [file plants-14-03183-s001.zip › plants-3916012-supplementary.pdf]

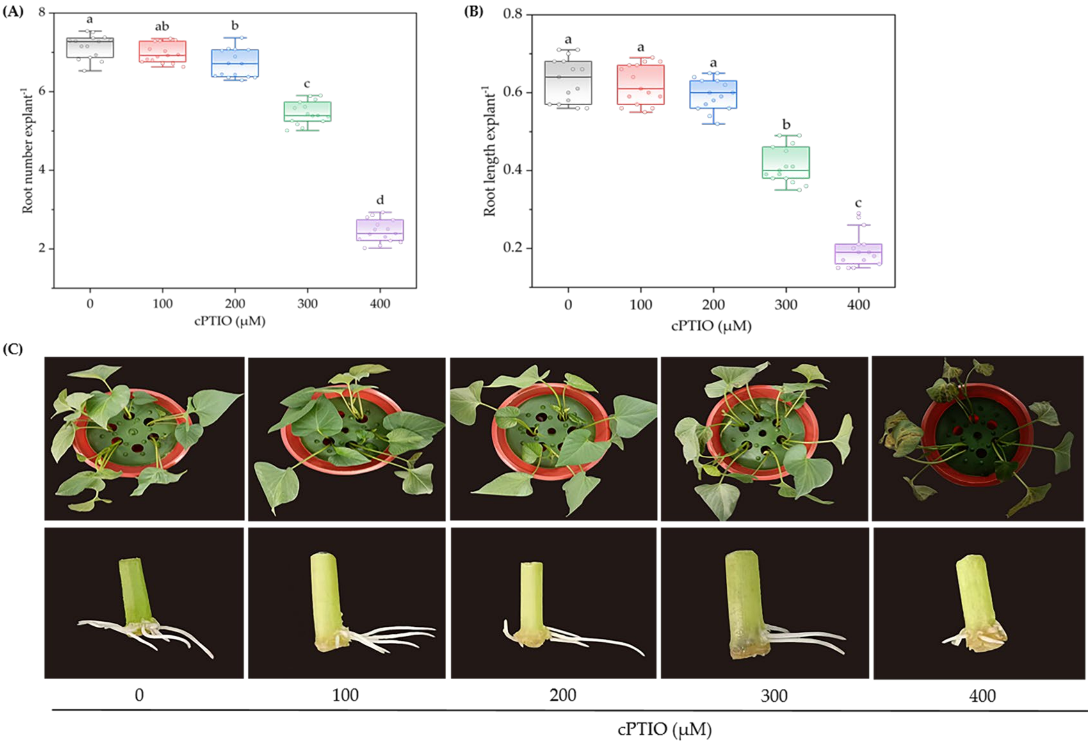

Figure S1. Effects of NO scavenger cPTIO on adventitious root development from sweetpotato cuttings. The primary root system was removed from the hypocotyls of 45-day-old sweetpotato seedling. Cuttings were incubated for 7 d with SNP at 0, 100, 20, 300 and 400 μM, as indicated. Shown are boxplots of root number (A) , root length (B) for five treatments (n=3, with 5 explants per replicate). In each plot, the central line represents the median, boxes indicate interquartile ranges, and whiskers show data spread. Different lowercase letters above the boxes indicate that the difference among treatments is significant ($p < 0.05$) based on one-way ANOVA followed by Duncan's multiple range test. Photographs (**c**) were taken after 7 d of treatments.
